# Does mHealth influence community health worker performance in vulnerable populations? A mixed methods study in a multinational refugee settlement in Uganda

**DOI:** 10.1371/journal.pgph.0002741

**Published:** 2023-12-29

**Authors:** Michael T. Wagaba, David Musoke, Arthur Bagonza, John B. Ddamulira, Christine K. Nalwadda, Christopher G. Orach

**Affiliations:** 1 Department of Community Health and Behavioral Sciences, School of Public Health, College of Health Sciences, Makerere University, Kampala, Uganda; 2 Department of Disease Control and Environmental Health, School of Public Health, College of Health Sciences, Makerere University, Kampala, Uganda; PLOS: Public Library of Science, UNITED STATES

## Abstract

Community Health Workers (CHWs) provide healthcare in under-served communities, including refugee settlements, despite various challenges hindering their performance. Implementers have adopted mobile wireless technologies (m-Health) to improve the performance of CHWs in refugee settlements. We assessed the CHWs’ performance and associated factors in a multi-national refugee settlement, operating mHealth and paper-based methods. This cross-sectional study employed quantitative and qualitative data collection methods. Data for 300 CHWs was collected from implementing partners’ (IPs) databases. Nine focus group discussions (FGDs) with the CHWs and community members, two in-depth interviews (IDIs) with CHW leaders, and eight key informant interviews (KIIs) with six IPs and two local leaders were conducted. The qualitative data were analysed thematically using AtlasTi version 9 while the quantitative data were analysed at the univariate, bivariate and multivariable levels using Stata version14. The study found that only 17% of the CHWs performed optimally. The factors that significantly influenced CHW performance included education level: secondary and above (APR: 1.83, 95% CI: 1.02–3.30), having a side occupation (APR: 2.02, 95% CI: 1.16–3.52) and mHealth use (APR: 0.06, 95% CI: 0.02-.0.30). The qualitative data suggested that performance was influenced by the number of households assigned to CHWs, monetary incentives, adequacy of materials and facilitation. Particularly, mHealth was preferred to paper-based methods. Overall, the CHWs’ performance was sub-optimal; only 2 in 10 performed satisfactorily. The main factors that influenced performance included the level of education, use of mHealth, having another occupation, workload and incentivisation. CHWs and IPs preferred mHealth to paper-based methods. IPs should work to improve refugee settlement working conditions for the CHWs and adopt mHealth to improve CHW performance.

## Background

Community health workers (CHWs) have been known to bridge the gap between under-served households and the formal health system by increasing access to Primary Health Care (PHC) [1]. CHWs are essentially part of the community that selects them, profoundly understand the community culture and language, have a shorter duration of training than health professionals and whose primary aim is to provide appropriate health services to the community [2, 3]. In the past two decades, community health workers have notably improved access to healthcare and uptake of health services in the African population [1]. Studies indicate that the impact of CHWs is more appreciable in vulnerable and resource-limited settings such as refugee settlements [4]. CHWs considerably enhance the sustainability, cost-effectiveness, and cultural acceptability of health programs and services in refugee communities, which is crucial in societies characterized by poverty, diverse cultures, and psychological trauma [5, 6].

However, CHWs usually face numerous challenges while executing their duties, affecting their performance and retention [7–11]. These challenges include poor financial incentives and facilitation [12–18], and inadequate support from the health system and beneficiary communities [6, 17–20]. Furthermore, an overwhelming workload, including the CHWs’ assigned tasks and side occupations, and low education levels (common in refugee communities), significantly lower the productivity, quality of service and overall performance of the CHWs [10, 11, 21–24].

Owing to the performance challenges, mHealth has been adopted into the CHW program [25–27]. mHealth is the use of mobile wireless technologies for public health surveillance and promotion [28]. Generally, studies on the adoption and advancements in mHealth show a positive correlation with improved healthcare practice and service delivery [29–31]. Similar correlations have been noted between mHealth and the performance of CHWs in low and middle-income countries. mHealth has been linked to improvements in various CHWs functions including community health surveillance, service delivery and data quality [27, 32–37]. However, using these technologies by CHWs presents challenges including loss of mHealth gadgets, weak technical support and poor network or internet access, hindering optimal performance [27, 33, 38, 39]. Nonetheless, there is limited evidence regarding the effectiveness of mHealth on the performance of the CHWs in conflict affected settings such as refugee settlements.

Uganda hosts the largest refugee population in Africa and is the third largest refugee hosting country, worldwide. Over 1,500,000 refugees are distributed in 13 settlements within Uganda [40], and the number continues to grow due to the civil wars and hostilities in the great lakes region countries. By February 2022, Kyaka II refugee settlement had more than 135,000 refugees and asylum seekers from over seven nationalities. The settlement is mixed with Ugandan nationals [41]. The constant influx of refugees from the Democratic Republic of Congo (DRC) and Rwanda exacerbates the health services delivery demands and challenges [42]. However, the settlement has only three Health Center III facilities and six medical outposts, serving over 141,000 people (including the host communities) [43]. This extremely low professional healthcare workers to population served ratio creates a great demand and justification for shifting the task of health service delivery to CHWs in such vulnerable settings.

Community health workers are expected to perform various tasks in the settlement including household visits to record the community’s vital statistics and surveillance; immunization, referrals, ante/postnatal care promotion, basic disease management activities and community mobilization. CHW performance is assessed by the monthly, quarterly and annual report analyses premised on the fulfilment of their roles and obligations [44]. Evidence on performance of CHWs from previous studies has been premised on measuring CHW functions including household visits conducted, referrals to facilities made, childhood vaccination coverage, supervisions and trainings attended [44, 45]. Evidence indicates that the performance of the CHWs in the national health care delivery system is suboptimal, ranging from 11% to 40%, [45–47]. However, very few such studies have been carried out in (the more vulnerable) refugee settlements where the CHW approach to healthcare delivery would address cultural appropriateness and protection concerns [5].

Nevertheless, mHealth was introduced to Kyaka II by the health implementing partner to improve CHW performance. CHWs were provided with smart-mobile phones onto which an online data collection app (Kobo Collect) with a GPS locator were uploaded. This enabled them to collect and report data during the household visits, instead of using registers and counter books. The influence of mHealth on the crucial CHW services delivered to the refugee communities was assessed. We examined CHWs’ performance and associated factors in a multi-national refugee settlement, implementing mHealth and the conventional paper-based methods. Using the technology acceptance model (TAM), we evaluated the refugee CHWs’ effective use of mHealth to improve performance [48]. TAM was the more appropriate model to evaluate feasibility and perceived usefulness of mHealth among the end-users and non-users [49].

## Methods

### Ethics statement

The study protocol was approved by the Higher Degrees, Research and Ethics Committee of Makerere University School of Public Health (Protocol number: 059). Written Informed Consent was sought from each of the study participants prior to their participation in the interviews. No identifying information was collected from any of the study respondents.

### Study design and setting

This was a cross-sectional study employing concurrent quantitative and qualitative methods of data collection and analysis. A mixed methods approach was adopted to foster a comprehensive examination of the study subject within this unique but under-studied refugee settlement context. Using mixed methods was a better approach than quantitative-only or qualitative-only methods when a single data source is not sufficient [50, 51]. The quantitative methods analysed secondary data to determine the CHWs’ performance levels and the associated factors. The qualitative methods explored the stakeholders’ perspectives on the CHWs’ performance determinants and on the use of mHealth in the refugee settlement.

The study was conducted in Kyaka II Refugee Settlement located in Southwestern Uganda. The settlement consists of nine zones and 26 villages hosting a population of an estimated 135,827 refugees and asylum seekers in 43,447 households. Most refugees are from the Democratic Republic of Congo (129, 367), Burundi (3,424) and Rwanda (3,263) [41]. This settlement was chosen because it consists of coexisting Ugandan and multi-national refugee communities, and CHWs employ paper-based and mHealth methods concurrently. The CHWs’ socio-demographics and functional reports are routinely updated in electronic databases. However, the paper-based data is backed up electronically using Epicollect and Microsoft excel files. Therefore, this ensured the reliability and credibility of the secondary data about CHWs who use both methods, enabling their assessment within the same context.

### Participants

The study aimed to use all records of the 308 refugee and national CHWs for 12 months. However, we included 300 CHWs’ performance records whose data was readily available. We considered that sampling would reduce the number of records, leading to the loss of information, data variability and power of the study. For the qualitative methods, we conducted 9 FGDs, 2 IDIs and 8 KIIs that sufficed to reach saturation. The two In-Depth Interviews (IDIs) were conducted with CHW leaders. The eight Key Informant Interviews (KIIs) were conducted with one Local council (LC) chairperson and one Refugee Welfare Councilor (RWC); two CHW supervisors—public health officers from the health Implementing Partners (IPs); the HCIII facility head and two medical outpost in-charges; and a UNHCR representative. The nine FGDs comprised the following categories: female refugee CHWs (1), Host CHWs (1), host community members (1); CHWs who use mHealth (1) and Paper-based (1) methods; refugee CHWs from the best (1) and worst (1) performing zones; the beneficiary refugee communities from the best (1) and worst (1) performing zones—based on the settlement’s records from the previous year; and. Participants were selected purposively based on their designations, knowledge and experience pertaining to CHW programs thus gathering rich data on policy, practice and management. However, all CHW records with less than 10% of the entries were not considered. Also, among the selected participants for qualitative data, those who did not give informed consent or were in a poor health state at the time of the study, were excluded.

### Data collection

The qualitative and quantitative data were collected concurrently. A data extraction tool was developed using Microsoft Excel to capture performance indicators, metadata and variables relevant to the study. The CHW monthly reports were obtained from the databases of the Implementing Partners (IPs) from 26^th^– 28^th^ July, 2022. Data of the CHWs who use mHealth was retrieved from the KoBo Toolbox database, while that from the paper-based model was collected from Epicollect and Microsoft Excel files where they had been backed up.

For the qualitative component, the identified KII and IDI participants were contacted via telephone between the 14^th^ and 20^th^ of July, 2022, while FGD participants were contacted two days prior to data collection, to schedule an appropriate time and venue. Overall, qualitative data was collected from 21^st^ July to 14^th^ August, 2022. All interactions with participants were face-to-face except for two KIIs which were conducted via telephone as they were not on site at the time of data collection. All the participants gave written informed consent to audio record the sessions. The reviewed literature informed the topics of discussion in the IDI, FGD and KII guides. Nevertheless, we further probed about any related concepts that emerged during the interviews. On average, the sessions lasted 63 minutes.

Prior to data collection, seven research assistants with prior research experience in the settlement were trained for three days on the key study concepts and to strictly observe the ethical considerations for vulnerable refugee populations. The tools were also translated into Swahili, a common language in East Africa, which was understood by all the FGD and IDI participants. However, all KIIs were conducted in English–the official language used by all government officials and IPs. The qualitative tools were pretested in Kaborogota—one of the zones that had not been selected to participate in the study—using two CHW FGDs, one Community FGD and four KIIs from IPs. Adjustments were made accordingly to improve the flow of the questions in the tools. The principal investigator supervised the data collection and management.

### Performance measurement

CHW Performance was either optimal or sub-optimal. Optimal performance was achieved by scoring well in at least four of the five indicators considered critical to the refugee settlement. Each CHW was assessed per selected indicator; an average score (over the 12 months) of at least 80% in the selected indicators qualified them as good performers. The indicators included: the percentage of monthly household visits conducted and reported of those assigned to each CHW over the 12 months; the proportion of children under five (U-5s) years screened for malnutrition (assessed per CHW catchment area over 12 months), using the total number of U-5s in each CHW’s catchment area as the denominator; the proportion of trainings attended over the 12 months, considering the total number of trainings scheduled for CHWs by the IPs in 2021 as the denominator; the completeness and coherence of CHWs’ reports on selected pertinent indicators over the 12 months, objectively scored by two IP public health officers who had closely supervised the CHWs; and the proportion of expected adverse medical condition referrals to health facilities over the 12 months guided by the health IPs. The settlement’s health IPs recommended a minimum of five referrals per month to be optimal, for each CHW. Previous CHWs’ performance measurement studies informed the selected performance indicators and variables [44, 45, 52], triangulated by information from pilot interviews with the settlement’s stakeholders (representatives of IPs, OPM, Health Facilities and CHWs) for context relevance. Indicators were chosen based on settlement-specific factors, including high stunting levels and a large refugee population with few CHWs.

### Data management and analysis

#### Data management

Quantitative data from the electronic datasets and the CHW registers was captured using Microsoft Excel, disaggregated into the paper-based and mHealth user datasets, and cleaned. The Excel datasets were imported into the Stata.14 software for analysis. The audio data was backed up daily as computer audio files and transcribed. The audios in Swahili were translated into English for uniformity and transcribed verbatim while iterating with notes taken during data collection.

#### Data analysis

Univariate analysis for data was conducted and reported as mean and standard deviation for continuous variables; and frequencies and proportions for discrete and categorical variables.

Bivariate analysis was conducted using Chi-square and Fisher’s exact tests to establish strengths of association between CHW performance and the predictor variables. We obtained prevalence ratios (PR) since the study found that the CHW performance level was 17%; and those from previous studies ranged between 11%–40% [45, 53]. Prevalence ratios are preferred to odds ratio (OR) when the dichotomous outcome (CHWs’ performance) is greater than 10% [54–57]. To qualify for multivariate analysis, we considered associations of a p-value less than 0.25 at bivariate analysis. At the multivariate level, the modified (robust variance) Poisson regression model was used [58].

The transcripts in English were collectively proofread by two experienced, independent researchers to ensure coherence with audio recordings. We developed a codebook inductively with codes having clear definitions, and later developed categories and themes through a consensus process. The analysis was thematic; we used AtlasTi. version 9 software for coding and managing query reports. The qualitative narratives were used to complement the quantitative findings [59]; emphasized by illustrative non-attributable quotes from participants.

## Results

Both qualitative and quantitative data were analyzed simultaneously, and the results were compared for complementarity. The characteristics and overall performance of CHWs were presented quantitatively, while the perceptions on the use of mobile health (mHealth) were presented qualitatively. The factors associated with performance were presented both qualitatively and quantitatively. All quantitative results agreed with the qualitative except for the impact of mHealth use on CHW performance.

### Participant characteristics

The mean age of the participants was 34.3 (SD±9.2) years. Most of the participants, 63.0% (189/300) were aged ≤ 35 years, two-thirds, 67.7% (203/300) were male and the majority, 87.3% (262/300) were refugees. Nearly two-thirds, 61.0% (183/300) had a secondary level of education, 63.7% (191/300) had another occupation besides CHW work and 68.3% (205/300) owned mobile phones (Table 1).

**Table 1:** Socio-demographic and work characteristics of the CHWs.

Variable	Category	Frequency (n = 300)	Percentage (%)
**Age (mean = 34.3, SD = 9.2) years**	≤ 35	189	63.0
	> 35	111	37.0
**Sex**	Female	97	32.3
	Male	203	67.7
**Education level**	None	2	0.7
	Primary	104	34.7
	Secondary	183	61.0
	Tertiary	11	3.7
**Marital status**	Single	37	12.3
	Married	254	84.7
	Divorced	9	3.0
**Nationality**	National	38	12.7
**Duration of work as CHW (mean = 4.0 SD = 2.3) years**	Refugee	262	87.3
	0–3	146	48.7
	4+	154	51.3
**Other/side occupation**	None	109	36.3
	Has other occupation	191	63.7
**Mobile phone ownership**	No	95	31.7
	Yes	205	68.3
**Mode of reporting**	m-Health	127	42.3
	Paper-based	173	57.7

### Overall performance of the CHWs

**Fig 1:**
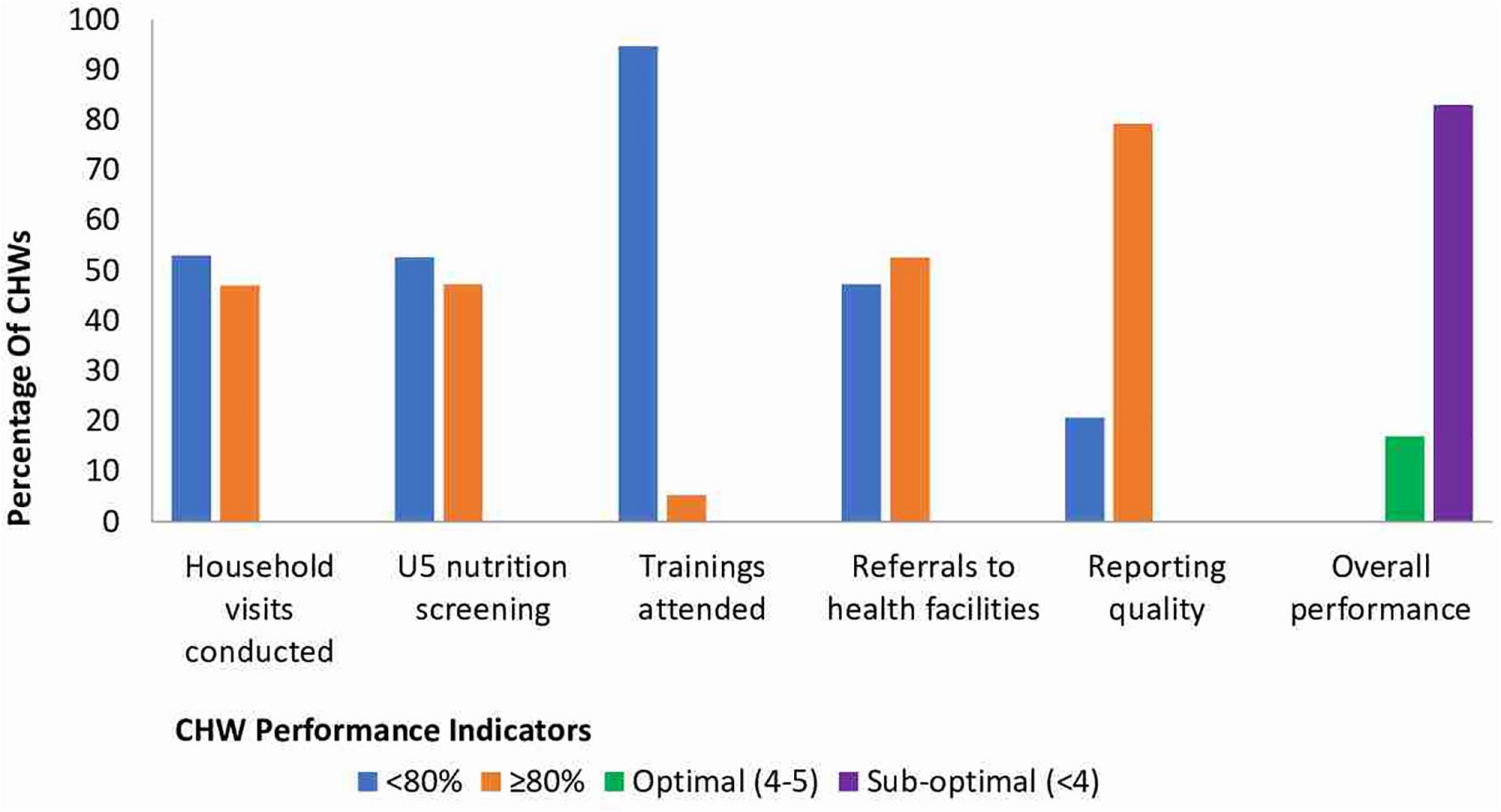
Performance of CHWs in Kyaka II refugee settlement.

Only 17.0% (51/300) of the CHWs exhibited optimal performance. Out of the five indicators, trainings attended had the lowest score, with only 5.3% (16/300) attending the required scheduled trainings. Conversely, reporting quality had the best score at 79.3% (238/300), with all mHealth users scoring 100% on this indicator. These scores are aggregated across both groups using paper-based and digital methods (Fig 1).

### Factors associated with CHW performance

The CHW performance determinants are categorized under facilitators and barriers and were quantitatively analysed at bivariate and multivariable levels (Table 2).

**Table 2:** Determinants of the performance of CHWs in Kyaka II refugee settlement.

*Variable*	*Performance level*		*CPR (at 95% CI)*	*P-values*	*APR (at 95% CI)*	*P-values*
	Optimal (N = 51)	Sub-optimal (N = 249)				
	F (%)	F (%)				
***Age (years)***						
*≤ 35*	32 (62.8)	157 (63.1)	1			
*> 35*	19 (37.3)	92 (36.9)	1.01 (0.60–1.69)	0.967		
***Sex***						
*Male*	33 (64.7)	170 (68.3)	1			
*Female*	18 (35.3)	79 (31.7)	1.14 (0.68–1.92)	0.619		
***Education level***						
*Primary and below*	10 (19.6)	96 (38.5)	1		1	
*Secondary and above*	41 (80,4)	153 (61.5)	2.24 (1.17–4.29)	**0.015** *	1.83 (1.02–3.30)	**0.043** *
***Marital status***						
*Single*	5 (9.8)	32 (12.9)	1			
*Married*	44 (86.3)	210 (84.3)	1.28 (0.54–3.03)	0.571		
*Divorced*	2 (3.9)	7 (2.8)	1.64 (0.33–7.16)	0.508		
***Nationality***						
*Refugee*	50 (98.0)	212 (85.1)	1		1	
*National*	1 (2.0)	37 (14.9)	0.14 (0.02–0.97)	**0.047** *	0.80 (0.14–4.53)	0.802
***Duration worked as a CHW***						
*0–3*	26 (51.0)	120 (48.2)	1			
*4+*	25 (49.0)	129 (51.8)	0.91 (0.55–1.50)	0.717		
***Other occupation***						
*None*	12 (23.5)	97 (39.0)	1		1	
*Has other occupation*	39 (76.5)	152 (61.0)	1.51 (0.85–2.66)	0.157	2.02 (1.16–3.52)	**0.013** *
***Owns mobile phone***						
*Yes*	23 (45.1)	182 (73.1)	1		1	
*No*	28 (54.9)	67 (26.9)	2.63 (1.60–4.31)	**<0.001** *	1.12 (0.70–1.78)	0.646
***Households allocated***						
*60 and below*	8 (15.7)	17 (6.8)	1		1	
*Above 60*	43 (84.3)	232 (93.2)	0.49 (0.26–0.92)	**0.027** *	0.61 (0.34–1.09)	0.094
***Mode of reporting***						
*Paper-based*	49 (96.1)	124 (49.8)	1		1	
*mHealth*	2 (3.9)	125 (50.2)	0.06 (0.014–0.23)	**<0.001** *	0.06 (0.02–0.30)	**0.001** *

*Statistically significant if p-value < 0.05 at 95%CI

The bivariate analysis revealed that the performance level of refugee CHWs was 7.14 times better than that of CHWs from the host communities (CPR = 0.14; 95% CI: 0.02–0.97).

### Facilitators to CHW Performance

The study found that CHW performance was positively influenced by high education levels and a side occupation, consistent across both quantitative and qualitative components.

#### Education level

Having attained secondary level education and above was positively associated with the level of performance. CHWs with secondary education or higher performed 2.24 times better than those with primary education or less. (CPR = 2.24; 95% CI: 1.17–4.29). This association was substantiated at multivariable analysis. (APR = 1.83; 95%CI: 1.02–3.30). Most KIIs and both IDIs reported that CHWs with higher education levels easily attained knowledge and skills, improving performance. A higher education level was also linked with improved community integration skills, improving the likelihood of better performance.

*"Education level*, *we find that the people who are much more educated will understand most of these things that we need faster than those who are not*.*”*

*(***Key Informant_Health Professional**)

#### Side occupations

CHWs who had side occupations performed 1.51 times better than those who did not have any other occupation. (CPR = 1.51; 95% CI: 0.85–2.66); the association was significantly stronger (twice better) at multivariable analysis (APR = 2.02; 95%CI: 1.16–3.52). Some key informants mentioned that CHWs with other occupations often performed better because they had an intrinsic result-oriented self-drive. Moreover, income from additional occupations covered coordination expenses, a crucial aspect of achieving optimal performance.

*"They will not look at VHT work as a source of income but like I said*, *they are those who are self-driven and are respected by the community (…) he is able to buy his data bundles and submit reports on time*, *able to give weekly reports*, *he is available*, *he can easily call to link someone*, *he can call for an ambulance; so yeah people are always coming to him*. *Because of the extra income*, *he is able to buy his own data and give extra services fully and entirely"*

(**Key Informant_Health Professional**).

The qualitative component of the study revealed other factors that positively influenced CHW performance beyond those examined quantitatively. The factors included; personal conviction, optimal coordination and supportive supervision.

#### Individual conviction and dedication

Most FGD participants and both IDIs reported that CHWs were intrinsically motivated by their conviction and devotion to improving the living conditions of their communities. They perceived executing their roles well as the uttermost obligation to their communities that would prevent further suffering and death.

*"Honestly speaking*, *volunteering is very difficult; if you are not devoted*, *you cannot be used as a VHT*. *What made us decide to do this was because we saw our mothers and children suffering; today there was death here and tomorrow*, *there*. *So*, *we decided to stand up and support our community"*

(**Focus Group Discussion_CHWs_Mixed**)

#### Coordination and supportive supervision

Some KIIs and FGDs noted that effective coordination and supportive supervision from the IPs and health facilities enhanced the CHWs’ skills, confidence and cohesion, enabling them to function adeptly.

*"To a bigger extent*, *we [IPs] have been a support system*. *This support system is the one that is helping them [CHWs] to a bigger extent to work well"*.

(***Key Informant Interview_Health facility In-charge***).

*"What can make me leave this VHT work is if I had a bad VHT leader*, *but I thank God that we have a good leader*. *So even if your heart was breaking*, *you hang in there"*.

(**Focus Group Discussion_CHWs_Female**).

### Barriers to CHW performance

#### Using mHealth

Using mHealth for reporting negatively affected the CHWs’ performance. The CHWs that used the paper-based methods performed almost 17 times better than those that used mHealth at bivariate (CPR = 0.06; 95% CI: 0.014–0.23) and multivariable (APR = 0.06; 95%CI: 0.02–0.30) analysis. However, the qualitative findings indicated a discrepancy with the quantitative findings. Most FGD, IDI and KII participants expected that mHealth would enhance CHW performance. They believed that using mHealth methods to collect and report health data increased data quality and the productivity or efficiency of the IPs and CHWs.

*"But I think mHealth would be performing better than the paper-based because if I have given a referral*, *I indicate it there in the mHealth*, *everything is tallying; the home visits*, *its auto calculated; the accuracy is better; things are controlled*. *But for paper-based*, *someone can forget his referral*, *and one would say he is not performing"*.

(**Key Informant _UNHCR**)

#### Number of households assigned to a CHW

Having many households to cover increased the workload, negatively impacting CHW performance. Bivariate analysis showed that CHWs with over 60 households had performance levels half that of their counterparts with 60 or fewer households (CPR = 0.49; 95% CI: 0.26–0.92). To this effect, the findings from most FGDs, KIIs and both IDIs concurred that CHWs were assigned greater tasks than they could effectively accomplish. CHWs complained of the high number of households assigned to them and the vast distances covered to reach each household. They were also overwhelmed by the bulk of monthly reports required by various implementing partners.

"*It [the workload] is too much because reporting ten households in a day is too much*. *Households in our villages are scattered*, *so you have to move from house to house"*.

(**In Depth Interview_Refugee CHW**)

“Their [CHWs] plans are usually disorganized by the many assignments from IPs”

(**Key Informant_Medical outpost**)

Other barriers to CHW performance emerged from the qualitative component of the study, including inadequate incentivization and poor facilitation of the CHWs.

Most FGD, KII and both IDI participants mentioned that inappropriate financial incentives demoralized the CHWs. Despite of innovations to enhance performance such as mHealth, low financial incentives, and the occasional lateness of the payments further reduced the CHWs’ morale to perform optimally.

*"It is affecting us because it delays the payment*, *and yet it is already small*. *The money is small*, *but if they keep paying us on time*, *it would be good*.*"*

(**In Depth Interview_Refugee CHW**)

Several participants identified poor facilitation of CHW functions, as a key factor that both CHWs and the communities they served agreed on, to have frustrated and deterred CHW performance efforts. There was reduced productivity when the IPs did not provide CHWs with the appropriate mHealth materials and medical supplies. to perform optimally.

*"How can they send soldiers to war without guns*? *How can they*? *Another thing is to give us transport refund once they call us for training*. *What they give us usually is very small"*

(**FGD_CHWs_Best performing zone**).

These challenges were linked to the abrupt change of IPs, each with different terms of reference. Such impromptu changes frustrated the CHWs’ operations and relationship with the IPs.

*"There was one organization that managed affairs in this camp*, *DRC knew how to deal with refugees*, *but now they are out"*.

(**FGD_CHWs_Worst performing zone**).

### Perceptions and experiences of the settlement stakeholders with using mHealth

Findings from the Community Health Worker FGDs and KIIs indicated a preference for the mHealth method over the paper-based one. The participants raised various reasons, including portability, reduced workload, improved data reporting and validity (reduced data falsification), improved data storage and safety, and the ability to report to all pertinent IPs promptly and satisfactorily.

*"I prefer the phone because since we serve like seven IPs*, *you can send one report to all instead of seven long reports repeating the same thing*. *Furthermore*, *the bag is too heavy*, *it breaks the back so*, *if you have a phone*, *you are better off*. *Today if I am asked to choose between the tablet and the book*, *I will throw the book far away"*

(**FGD_CHWs_mHealth users**).

*“The phone will reduce the work load and protect the data*. *We will not be carrying pens*, *MUAC tape and books in the hands*. *In my area there is a large wetland that I have to cross yet I have no bag to carry my things*, *sometimes my materials fall and get dirty or soaked”*

(**FGD_CHWs_Best performing zone**).

Using mHealth also gave the CHWs a sense of achievement, respect and power among the community members they served. Similarly, the female CHWs found mHealth more efficient, saving them more time to attend to their domestic duties. Consequently, the CHWs gained more morale of the CHWs to serve diligently.

*"A VHT must have a smartphone and if you give us*, *I would move from "kapeesa" [analogue] phone to the smart one*. *People can know that VHTs are developing and so they will respect us*.

(**In Depth Interview_Host CHW_Female**).

*“but that phone will help us to manage time*, *because when you finish your work from the field and send*, *you will get relief that now you can do house chores without disturbance”*.

(**FGD_CHWs_Female Group**).

Some key informants asserted that rolling out mHealth was inevitable based on experiential evidence and CHWs’ consensus.

*"But in my belief*, *we are meant to go digital*. *Based on what I have seen*, *what I have experienced*, *and the feedback I have gotten from the VHTs*. *It is beyond the way to go*, *and given how our world is moving*, *we have no other way"*

(**Key Informant Interview _Public Health Assistant_Male**).

However, mHealth use presented challenges that hindered CHW performance. Some KIIs were concerned with the program’s sustainability, citing potential theft, loss, sale or damage of the smartphones given the dire socioeconomic status in this settlement.

"*I will be very honest*, *it [mHealth]hasn’t been very sustainable (…)*. *Basically*, *when someone loses their reporting tool*, *their smartphone or their tablet*, *it will affect the drop out since*, *you can no longer work with the team because your mode of reporting is no more*. *So*, *now*, *with these gadgets*, *there are security issues; someone will steal it; it will get spoilt; it will break"*

(**Key Informant_Public Health Assistant**)

Furthermore, some KIIs and FGDs revealed some mHealth usability challenges, including illiteracy levels which hindered CHWs from readily adapting to mHealth use, and the limited internet connectivity and electricity sources in several areas within the settlement, frustrating the reporting.

*"We are trying to roll it out*, *but the issue was charging the phone*, *you know most of these people do not have power (…) Also*, *the issue of VHTs being unable to use the phone because they are illiterate*, *so it will take a long time to train and bring them on board"*

(**Key Informant_UNHCR**).

*“The challenge with the phone*, *sometimes you find there is no network where you have reached visiting that family*, *there is no network and it first disturbs you”*

(**FGD_CHWs_Mixed Host Communities**).

## Discussion

The study assessed the performance and associated factors, of CHWs who use mHealth and paper-based methods in a multi-national refugee settlement in Uganda. Performance was rated either optimal or sub-optimal upon scoring more than 80% in at least four of the five selected indicators, derived from CHW roles pertinent to the settlement. The study used the mixed methods approach to investigate the impact of mHealth tools and associated factors on the performance of CHWs. The qualitative component was particularly important in comprehending the context of resource-limited, vulnerable refugee communities. Additionally, since certain variables were not present in the dataset for quantitative analysis, they were examined qualitatively to enhance the study’s validity.

At the program level, only 17% of the CHWs in Kyaka II refugee settlement performed optimally. Previous studies carried out in non-humanitarian settings in Uganda found CHW performance ranging between 11%–40% [45–47]. The worst performed indicator was the proportion of trainings attended, which is critical for the functioning of CHWs. Most CHWs lacked adequate training due to the inequitable selection criteria for training and logistical constraints. Particularly, inadequate training and facilitation for the mHealth users in the settlement could have created performance inequalities [33]. Training is a critical determinant of CHW performance outcomes [6]. However, the indicator on the quality of reports had the best performance score, which was closely linked to the use of mHealth. All mHealth users scored maximum points (100%) due to the programmed quality checks within the mHealth tools preventing falsifications and omission of data. This finding agreed with a previous study positing that mHealth use improved data validity and reliability [33]. On the other hand, despite the introduction of mHealth technologies, CHW performance in the settlement remained sub-optimal, possibly due to sociological, resource and technical constraints [33, 38, 39]. Improving training and facilitation of CHW mHealth initiatives could improve the program outcomes in refugee settings.

At the individual CHW level, the study revealed several factors were associated with the sub-optimal CHWs’ performance. Firstly, CHWs with a higher level of education were significantly more likely to perform better because they were trainable, especially in health-related matters. Various studies allude to the positive association between the CHWs’ education level and performance [23, 24], and mHealth adoptability [60]. Furthermore, the highly educated CHWs quickly bonded with the professional health workers and were more frequently selected for training, including on mHealth use, than their counterparts. These acquired prerogatives increased their access to knowledge and resources to facilitate better execution of their functions, enabling them to perform better than their less educated counterparts.

The study also found that CHWs with other occupations performed significantly better than their counterparts without other occupations because of the extra income that they got. This was attributed to the heightened sense of accomplishment and contentment, which propelled the CHWs to serve their communities better, with fewer grievances to counter their innate drive to serve. However, these findings contradicted the reviewed literature, which postulated that other occupations distracted CHWs from maximally attending to their roles leading to sub-optimal performance [8, 9]. This contradiction could result from the stark dire economic status of the CHWs in the refugee settings that hinders optimal performance. The income from other jobs helped CHWs pay for transportation, communication, and internet expenses. These were crucial for using mHealth effectively, attending training, coordinating tasks, and referring severe cases to health facilities, all critical determinants of CHWs’ performance. Transport and telecommunication costs are not catered for by the implementing partners, implying that CHWs, especially the mHealth users, who could not facilitate themselves risked performing sub-optimally.

Using mHealth was significantly associated with sub-optimal performance in the refugee settlement. This finding differed from previous studies that showed strong correlations between mHealth use and improved CHW performance by simplifying and reducing the workload [27, 31, 35, 36]. These equivocal findings could be due to the refugee settlement operational settings and methodological discrepancies during analysis. There were limited resources to replace the mHealth gadgets in the case of loss, sale or damage, given the socioeconomic setting of the settlement. These constraints frustrated the routine data collection and reporting by the mHealth cohort, yet, data from both cohorts were indistinguishably analyzed over the entire 12 months. Despite the sub-optimal performance, the mHealth users found it more efficient as it reduced their workload, thus improving their productivity as postulated by previous studies. Improvements in the mHealth tool and implementation design could foster better performance.

The workload—the number of households assigned to each CHW—varied and was a key determinant of performance. Those with a larger number of households performed poorly because they could not traverse vast distances with unfriendly terrain and weather to cover all the assigned households. This finding agreed with previous studies in normal and humanitarian settings that found that the greater the CHWs’ workload, the poorer they performed [10, 11]. The average number of households assigned to the CHWs in the settlements is 100, significantly exceeding the 30 households recommended by the Ministry of Health [44]. Moreover, both quantitative and qualitative findings depicted that more workload on CHWs hindered their performance. However, the qualitative data suggested that using mHealth lessened the workload and improved their productivity [27, 32, 38]. Despite the availability of technologies like mHealth, an optimal number of households should be allocated to improve CHW service delivery.

In addition, the qualitative findings were in agreement with previous studies postulating that financial and non-financial incentives were crucial determinants of the CHWs’ performance. In Kyaka II refugee settlement, late or total removal of payments significantly demoralized the affected CHWs, leading to poor performance and dropout rates as depicted in previous studies [14, 15]. Similarly, the inadequate provision of materials and facilitation to the CHWs reduced their morale and ability to serve their communities to the extent of being rejected by their communities for having nothing to offer. The reviewed literature showed similar findings positing low CHW performance resulting from poor facilitation [16] and the lack of support from the beneficiary community [17, 18]. The insufficient facilitation hindered the optimal use of mHealth gadgets contributing to the poor performance of the CHWs who used mHealth [33, 38]. Poor facilitation could result from the resource-constrained nature of the refugee settlement, aggravated by the constant influx of refugees, which increased pressure on the supplies. Literature in similar settings showed that the increased demand for supplies and materials among Internally Displaced Persons (IDPs) negatively impacted CHW performance [61].

The views of most stakeholders indicated a preference for mHealth to paper-based methods for data collection and reporting because it reduced their workload and was more efficient in meeting the targets set by the various Implementing Partners. Previous studies showed similar findings with stakeholders considering mHealth as pivotal to improving CHW performance [33, 35, 36, 38]. Generally, stakeholders expressed interest in rolling out digital methods (m-health and digital payments). However, hindrances threatened their effectiveness in refugee settings, such as inadequate training owing to an essentially illiterate community and unsustainable financial and technical constraints. It would be prudent to design digital methods that would be feasible and more effective in refugee settlement contexts.

### Study limitations and strengths

The study had some limitations. These included the shortfalls of secondary data, including inadequate information on some variables pertinent to CHW performance including financial incentives, supervision and data quality, which could have affected the validity of the findings. Being a pilot program, the mHealth users could have lacked the proficiency to use the tools due to inadequate training and low education levels, giving inaccurate findings on the effect of mHealth. Furthermore, selecting a few among the various indicators for measuring CHW performance could have compromised the validity of the findings in other settings. In addition, social desirability bias could have compromised the validity of the qualitative responses. Supervisors (IPs) often influence the opinions of their subordinates. As such, the responses of the CHWs may have been biased towards the interests of the IPs.

However, these shortfalls were controlled by rigorous qualitative tools, source triangulation, comprehensive probing, assurance of confidentiality and utmost privacy of the IDI and FGD sites. In addition, the appropriateness of the variables for CHW performance assessment was informed by the IPs, availability of data and previous literature. Moreover, the secondary data was cleaned by the principal investigator and reviewed by two IP public health biostatisticians. These controls ensured the quality and validity of the data.

## Conclusion

The overall performance of CHWs was sub-optimal, with only about 2 in 10 performing satisfactorily. The IPs and CHWs preferred mHealth to paper-based methods because it lessened their workload and enhanced their efficiency. As health systems strive to digitize operations, end-users should be consulted and adequately trained on the appropriate mHealth modalities. Furthermore, service providers like electricity and mobile-network companies should be involved in the design and strategic implementation of these modalities. This would enhance the feasibility and sustainability of these mHealth modalities: proven to positively impact health service delivery in vulnerable settings.

## Supporting information

S1 Data. Stata dataset for the mHealth community health worker performance in Kyaka II refugee settlement (DTA)
